# Nonlinear Shrinkage Estimation of Higher-Order Moments for Portfolio Optimization Under Uncertainty in Complex Financial Systems

**DOI:** 10.3390/e27101083

**Published:** 2025-10-20

**Authors:** Wanbo Lu, Zhenzhong Tian

**Affiliations:** 1 School of Management Science and Engineering, Southwestern University of Finance and Economics, Chengdu 611130, China; 2 School of Statistics and Data Science, Southwestern University of Finance and Economics, Chengdu 611130, China

**Keywords:** nonlinear shrinkage, uncertainty, higher-order moment, complex systems, portfolio optimization

## Abstract

This paper develops a nonlinear shrinkage estimation method for higher-order moment matrices within a multifactor model framework and establishes its asymptotic consistency under high-dimensional settings. The approach extends the nonlinear shrinkage methodology from covariance to higher-order moments, thereby mitigating the “curse of dimensionality” and alleviating estimation uncertainty in high-dimensional settings. Monte Carlo simulations demonstrate that, compared with linear shrinkage estimation, the proposed method substantially reduces mean squared errors (MSEs) and achieves greater Percentage Relative Improvement in Average Loss (PRIAL) for covariance and cokurtosis estimates; relative to sample estimation, it delivers significant gains in mitigating uncertainty for covariance, coskewness, and cokurtosis. An empirical portfolio analysis incorporating higher-order moments shows that, when the asset universe is large, portfolios based on the nonlinear shrinkage estimator outperform those constructed using linear shrinkage and sample estimators, achieving higher annualized return and Sharpe ratio with lower kurtosis and maximum drawdown, thus providing stronger resilience against uncertainty in complex financial systems. In smaller asset universes, nonlinear shrinkage portfolios perform on par with their linear shrinkage counterparts. These findings highlight the potential of nonlinear shrinkage techniques to reduce uncertainty in higher-order moment estimation and to improve portfolio performance across diverse and complex investment environments.

## 1. Introduction

The mean–variance model proposed by Markowitz has long served as the cornerstone of modern portfolio theory, describing the allocation decisions of rational investors under uncertainty [1]. The framework assumes normally distributed returns and seeks to balance expected return against risk. However, subsequent studies have documented that financial returns deviate markedly from normality, exhibiting skewness, heavy tails, and excess kurtosis—features that reflect deep uncertainty in financial systems. Moreover, the quadratic utility implied by the mean–variance paradigm fails to capture realistic patterns of decreasing absolute risk aversion. In practice, investors are often willing to tolerate higher volatility in exchange for positively skewed and low-kurtosis returns [2]. Portfolios with such characteristics tend to offer greater potential for extreme gains [3] and stronger downside protection [4]. These observations have motivated the extension of portfolio theory to include higher-order moments as essential dimensions of risk and uncertainty.

Introducing higher-order moments into portfolio selection primarily follows three main directions [5]. The first extends the classical mean–variance paradigm through utility expansion models, where investor preferences are approximated by a Taylor series that incorporates third- and fourth-order terms. Early contributions demonstrated that portfolios with positive skewness and low kurtosis can yield higher expected utility and reshape the efficient frontier beyond the traditional trade-off between return and variance [6]. Building on these foundations, Harvey et al. [7] theoretically generalized the Markowitz model by embedding skewness and kurtosis directly into the optimization frontier, showing that higher-order risk preferences can substantially alter efficient portfolio sets. A second research stream emphasizes robust estimation of higher-order moments, addressing the severe dimensionality and instability of coskewness and cokurtosis matrices in large-asset universes. Representative studies include the nearest-comoment estimator with latent factors, the independent component approach for modeling higher-order dependence, and the parsimonious estimation framework [8,9,10], which maintains statistical precision while reducing computational cost. More recently, tensor-based and weak-factor approaches incorporate higher-order cumulant tensors to capture complex dependence and asymmetry in high-dimensional financial systems [11]. Furthermore, a third direction involves multiobjective and stochastic optimization frameworks that explicitly integrate higher-order moments into tractable portfolio design. These methods balance return, variance, skewness, and kurtosis within a unified optimization scheme, often employing successive convex approximation or parametric skew-t formulations for scalability [12,13]. Beyond the traditional higher-moment framework, several alternative methodological paradigms have recently emerged in portfolio optimization under uncertainty. Generative modeling approaches construct portfolios by simulating return distributions from latent processes; for example, Cheng and Chen propose a unified framework that combines generative forecasts with various optimization objectives and portfolio-blending strategies [14]. Reinforcement learning and deep reinforcement learning methods directly learn allocation policies or ranking mechanisms from market data. Alzaman, for instance, introduces a stock-ranking and matching model that exemplifies the shift toward adaptive and data-driven portfolio allocation [15].

Taken together, these developments underscore that the estimation of higher-order moments remains a critical bottleneck in extending portfolio theory beyond the mean–variance framework. To mitigate estimation noise and dimensionality, a growing body of work has turned to shrinkage estimation techniques. The linear shrinkage model proposed by Martellini and Ziemann [6] extends the constant-correlation framework [16] and the covariance shrinkage methodology [17,18]. This approach effectively stabilizes higher-moment estimates by reducing sampling noise and improving numerical conditioning. Subsequent developments generalized the shrinkage framework to multifactor environments, enhancing estimation precision and robustness in large-scale portfolio applications [19,20].

The pioneering work of Ledoit and Wolf marked a turning point in robust covariance estimation [17,18]. Their linear shrinkage estimator addressed the instability of the sample covariance matrix by shrinking eigenvalues toward a common mean, substantially improving estimation reliability in high-dimensional settings. Subsequent extensions introduced nonlinear shrinkage, in which eigenvalue shrinkage is adapted locally according to the empirical spectral distribution, thereby achieving further gains in accuracy and robustness [21,22,23]. In their most recent formulation, Ledoit and Wolf [24] proposed a local shrinkage scheme that smooths neighboring eigenvalues rather than enforcing a global shrinkage target, effectively filtering high-dimensional noise. Despite the extensive progress in covariance shrinkage estimation, relatively little attention has been devoted to extending these principles to higher-order moment structures, such as coskewness and cokurtosis tensors. Most existing studies remain confined to second-order dependence, leaving the problem of higher-order estimation uncertainty largely unresolved.

Building upon this line of research, this paper is the first to extend the nonlinear shrinkage estimation framework from covariance matrices to higher-order moment matrices, thereby broadening its applicability to complex systems and enriching methodologies for managing uncertainty in higher-order dependence structures. The proposed approach integrates factor models into the nonlinear shrinkage process to address the “curse of dimensionality” inherent in large-asset settings. Compared with conventional sample estimation, three-factor estimation, five-factor estimation, and multifactor linear shrinkage estimation, the proposed method demonstrates dual advantages: it minimizes mean squared error (MSE) and maximizes the Percentage Relative Improvement in Average Loss (PRIAL). Beyond statistical gains, the model delivers clear economic benefits: portfolios based on the nonlinear shrinkage estimator achieve higher annualized returns, lower kurtosis, higher Sharpe ratios, and reduced maximum drawdowns, providing stronger resilience to uncertainty in complex financial markets.

The primary contributions of the paper are as follows:

(1) It extends the nonlinear shrinkage framework from covariance matrices to higher-order moment tensors, addressing estimation uncertainty beyond the second moment.
(2) It integrates multifactor dimension reduction and tensor supersymmetry to ensure computational tractability in large-asset settings.
(3) It provides both theoretical justification and empirical evidence showing that the proposed estimator enhances portfolio robustness and investor welfare.

This paper fills this gap by generalizing nonlinear shrinkage to the realm of higher-order moment tensors within a multifactor framework, thereby providing a unified and theoretically grounded approach for reducing estimation noise in coskewness and cokurtosis estimation.

The remainder of this paper is organized as follows. Section 2 introduces the proposed nonlinear shrinkage method for higher-order moment modeling. Section 3 establishes its asymptotic properties. Section 4 reports the results of Monte Carlo simulations. Section 5 applies the method to portfolio construction. Section 6 conducts robustness checks on the portfolio results. Finally, Section 7 discusses the findings and outlines possible directions for future research.

## 2. Methodology

### 2.1. Representation of Higher-Order Moment Matrices

Let the number of assets be N and the number of observations be T. Denote by V, S, and K the covariance, coskewness, and cokurtosis matrices, respectively, of the asset return matrix R. The definitions of V, S, and K are as follows:

(1) $$\begin{array}{c} V=E\left[\left(R-\mu_{R}\right){\left(R-\mu_{R}\right)}^{T}\right]\\ S=E\left[\left(R-\mu_{R}\right){\left(R-\mu_{R}\right)}^{T}⊗{\left(R-\mu_{R}\right)}^{T}\right]\\ K=E\left[\left(R-\mu_{R}\right){\left(R-\mu_{R}\right)}^{T}⊗{\left(R-\mu_{R}\right)}^{T}⊗{\left(R-\mu_{R}\right)}^{T}\right] \end{array}$$

where R is an N×T  matrix of asset returns, and $\mu_{R}$ is an N×T  matrix of mean returns. The matrices V, S, and K correspond to the second-, third-, and fourth-order co-moment matrices of the R, with respective dimensions $N\times N,N\times N^{2}$, and $N\times N^{3}$. The operator ⊗ denotes the Kronecker product. The elements of V, S, and K can be expressed as follows:

(2) $$\begin{array}{c} \upsilon_{ij}=E\left[\left(R_{i}-\mu_{i}\right)\left(R_{j}-\mu_{j}\right)\right]\\ s_{ijk}\;=E\left[\left(R_{i}-\mu_{i}\right)\left(R_{j}-\mu_{j}\right)\left(R_{k}-\mu_{k}\right)\right]\\ k_{ijkl}\;=E\left[\left(R_{i}-\mu_{i}\right)\left(R_{j}-\mu_{j}\right)\left(R_{k}-\mu_{k}\right)\left(R_{l}-\mu_{l}\right)\right] \end{array}$$

Accordingly, S and K can also be equivalently expressed as:

(3) $$\;S=\left(S_{1}\left|\cdots \right|S_{N}\;\right),S_{u}=\left(\begin{array}{ccc} s_{u11} & \cdots & s_{u1N}\\ \vdots & \cdots & \vdots \\ s_{uN1} & \cdots & s_{uNN} \end{array}\right)\;K=\left(K_{11}\cdots K_{1N}\left|\cdots \right|K_{N1}\cdots K_{NN}\right)\;\;,K_{uv}=\left(\begin{array}{ccc} k_{uv11} & \cdots & k_{uv1N}\\ \vdots & \cdots & \vdots \\ k_{uvN1} & \cdots & k_{uvNN} \end{array}\right)$$

It should be noted that V, S, and K can be regarded as flattened representations of spatial tensors in Euclidean space. From the expressions of higher-order moment matrices, it is evident that as the order increases, the number of parameters to be estimated grows exponentially with the number of assets. This exponential growth not only imposes a heavy computational burden but also amplifies estimation uncertainty. This highlights the intrinsic challenges of modeling complex financial systems characterized by nonlinear dependence and heavy-tailed distributions. When higher-order moment matrices are rank-deficient or incomplete, traditional estimation methods often fail to provide stable and reliable results. Therefore, when dealing with higher-order co-moment estimation, it is imperative to adopt methods that can reduce the number of parameters to be estimated.

Supersymmetry in higher-order moment tensors implies that the tensors remain invariant under permutations of their indices. For instance, the third-order co-moment $E[x_{i}x_{j}x_{k}]$ is identical regardless of whether the indices are ordered as (i,j,k),(j,i,k), or (k,j,i). From a practical perspective, supersymmetry makes higher-order moment estimation more parsimonious and computationally efficient, which is essential in large-asset settings. By eliminating redundant parameters, it also reduces estimation noise and enhances the stability of portfolio optimization. This is particularly valuable for applications involving higher-order risk measures, such as skewness- or kurtosis-adjusted portfolio selection. For example, in the case of the third-order coskewness tensor, an unconstrained formulation would require estimating $N^{3}$ parameters. By applying supersymmetry, the number of unique elements is reduced to N(N+1)(N+2)/6, which greatly alleviates the curse of dimensionality. Table 1 reports the dimensionality reduction effect of supersymmetry on the number of parameters to be estimated in higher-order moments.

The introduction of tensor supersymmetry ensures that identical statistical interactions among assets are treated equivalently, effectively reducing the number of unique parameters to be estimated. This structural constraint improves numerical stability without sacrificing model flexibility.

In this paper, we draw on the nonlinear shrinkage covariance estimation method [22,23,24] and the multifactor higher-order co-moment estimation approach [19] to develop a novel nonlinear shrinkage estimation method for multifactor higher-order co-moment matrices. This method effectively reduces the number of parameters to be estimated and mitigates estimation uncertainty. By locally adjusting eigenvalues, nonlinear shrinkage reduces noise and stabilizes higher-order moment estimation, making it particularly suitable for complex financial systems with high-dimensional interactions.

### 2.2. Factor Model Estimation of Higher-Order Moment Matrices

The return vector is assumed to follow a factor structure:

(4) $$R_{t}=\alpha +BF_{t}+\varepsilon_{t}$$

where $R_{t}={\left(R_{1t},\cdots ,R_{Nt}\right)}^{T}$ is the N-dimensional return vector, $F_{t}={\left(F_{1t},\cdots ,F_{Qt}\right)}^{T}$ is the Q-dimensional factor vector, $\varepsilon_{t}={\left(\varepsilon_{1t},\cdots ,\varepsilon_{Nt}\right)}^{T}$ represents idiosyncratic risks orthogonal to factors, $\alpha ={\left(\alpha_{1},\cdots ,\alpha_{N}\right)}^{T}$ denotes the intercept vector capturing unexplained excess returns, and B is the N×Q full-rank factor loading matrix. The model assumes that factors are mutually independent, residuals are cross-sectionally independent, and factors are independent of residuals. By decomposing returns into common factors and idiosyncratic components, the factor model reduces the dimensionality of higher-order moment estimation. This not only alleviates the curse of dimensionality but also mitigates estimation uncertainty by filtering out noise and isolating the key drivers of dependence in complex financial systems. The higher-order moment matrices can be decomposed as:

(5) $$V=BS_{F}B^{T}+\Delta_{\varepsilon }\;S=BG_{F}\left(B^{T}⊗B^{T}\right)+\Omega_{\varepsilon }\;K=BP_{F}\left(B^{T}⊗B^{T}⊗B^{T}\right)+\gamma_{\varepsilon }$$

where V denotes the covariance matrix (second-order moment), measuring return volatility, S represents the coskewness matrix (third-order moment), capturing return asymmetry, and K is the cokurtosis matrix (fourth-order moment), quantifying tail risk. $S_{F}$, $G_{F}$, and $P_{F}$ correspond to the covariance, coskewness tensor, and cokurtosis tensor of the factors, expressed in matrix form. Similarly, $\Delta_{\varepsilon }$, $\Omega_{\varepsilon }$, and $\gamma_{\varepsilon }$ are the covariance, coskewness, and cokurtosis matrices of the residual term ε. The diagonal elements of $\Delta_{\varepsilon }$ are $E[\varepsilon_{i}^{2}]$, with off-diagonal elements equal to zero. $\Omega_{\varepsilon }$ is a matrix in which all entries are zero except for the {i,j}-th element, where $\Omega_{\varepsilon ,ij}=E[\varepsilon_{i}^{3}]$ and  j is defined as j=(i−1)*N+1 for all i=1,⋯,N. The elements of $\gamma_{\varepsilon }$ take the following form:

(6) $$6\sum_{q=1}^{Q}B_{iq}^{2}\sigma_{F,q}^{2}\sigma_{\varepsilon ,i}^{2}+E\left[\varepsilon_{i}^{4}\right]\;\;\forall i=j=k=l\;3\sum_{q=1}^{Q}B_{iq}B_{lq}\sigma_{F,q}^{2}\sigma_{\varepsilon ,i}^{2}\;\;\forall i=j=k\neq l\;\sum_{q=1}^{Q}B_{iq}^{2}\sigma_{F,q}^{2}\sigma_{\varepsilon ,i}^{2}+\sum_{q=1}^{Q}B_{lq}^{2}\sigma_{F,q}^{2}\sigma_{\varepsilon ,i}^{2}+\sigma_{\varepsilon ,i}^{2}\sigma_{\varepsilon ,l}^{2}\;\;\forall i=j\neq k=l\;\sum_{q=1}^{Q}B_{kq}B_{lq}\sigma_{F,q}^{2}\sigma_{\varepsilon ,i}^{2}\;\;\forall i=j\neq k\neq l\;0\;\;\forall i\neq j=k\neq l$$

Let $\sigma_{F,q}^{2}$ and $\sigma_{\varepsilon ,i}^{2}$ denote the variances of the q-th factor and the i-th asset residual, respectively. Through tensor expansion, elements in the tensor are mapped to the matrix $\gamma_{\varepsilon }$. Specifically, the element at position {i,j,k,l} in the tensor is mapped to the {i,s} position in $\gamma_{\varepsilon }$, yielding $\gamma_{\varepsilon ,is}$, where $s=1+(j-1)*N+(k-1)*N^{2}+(l-1)*N^{3}$.

Although factor models rely on the assumption of independence between factors and residuals, this assumption may be overly restrictive in empirical financial settings. Nevertheless, a growing body of literature indicates that factor-model-based estimation procedures remain robust even when the independence assumption is relaxed. Bai and Ng [25] demonstrate that consistent factor estimation is achievable under weak cross-sectional and temporal dependence of residuals. Ledoit and Wolf [17,18] further show that shrinkage-based covariance estimators retain good performance even when residuals are not strictly orthogonal to factors. More recent studies on approximate factor models explicitly allow for weak correlations and establish asymptotic properties under such relaxed conditions [26,27].

From Equations (4)–(6), it can be seen that when the number of assets is large, due to the small number of factors and the over-symmetry of the residual tensor, the factor-model estimation of higher-order moments can significantly reduce the number of estimated parameters, effectively mitigating the “dimensionality curse” problem caused by excessive asset dimensions.

### 2.3. Nonlinear Shrinkage Estimation of Higher-Order Moment Matrices

Let Σ denote the N-dimensional positive-definite population covariance matrix, and S denote the sample covariance matrix. Its spectral decomposition is $S=U\Lambda U^{T}$, where $\Lambda =\mathrm{diag}(\lambda_{1},\cdots ,\lambda_{N})$ is the diagonal matrix of sample eigenvalues, arranged in ascending order, and $U=[u_{1},\cdots ,u_{N}]$ is the orthogonal matrix of corresponding eigenvectors. Equivalently, the sample covariance matrix can be expressed as $S=\sum_{i=1}^{N}\lambda_{i}\cdot u_{i}u_{i}^{T}$. We then construct a covariance matrix estimator of the form $\hat{\Sigma }=U\hat{\Lambda }U^{T}$, where $\hat{\Lambda }=diag({\hat{d}}_{1},\cdots ,{\hat{d}}_{N})$, and ${\hat{d}}_{i}=\hat{d}(\lambda_{i})$ denotes the estimated eigenvalue obtained by applying a shrinkage function $\hat{d}(•)$ to the i-th estimated eigenvalue $\lambda_{i}$.

To demonstrate the superior performance of higher-order moment shrinkage estimation in terms of estimation accuracy, as well as its applicability and stability under various loss functions, this paper considers three shrinkage functions under different loss functions: the Stein loss function (referred to as linear inverse shrinkage, LIS), the Frobenius loss function (referred to as quadratic inverse shrinkage, QIS), and the symmetric Kullback–Leibler loss function (referred to as geometric inverse shrinkage, GIS).

#### 2.3.1. Stein Loss Function

The Stein loss function is defined as

(7) $$L^{ST}\left(\Sigma ,\hat{\Sigma }\right)=\frac{1}{N}Tr\left(\Sigma^{-1}\hat{\Sigma }\right)-\frac{1}{N}logdet\left(\Sigma^{-1}\hat{\Sigma }\right)-1$$

where Tr(⋅) denotes the trace, det(⋅) denotes the determinant. The optimization solution for the loss function is given by:

(8) $$\;\arg\;\underset{\;\hat{\Lambda }}{\min}\;L^{\mathrm{ST}}\left({\Sigma ,U\hat{\Lambda }U}^{T}\right)\;\bar{\Lambda }=Diag\left({\bar{d}}_{1},\cdots ,{\bar{d}}_{N}\right)$$

The optimal estimator for Σ is given by $\bar{\Sigma }=U\bar{\Lambda }U^{T},$ where $\bar{\Sigma }$ is infeasible in practice because the population eigenvalues Σ embedded in ${\bar{d}}_{i}$ are unobservable. Stein [28] proposed an approximate estimator for the unobservable ${\bar{d}}_{i}$ as follows:

(9) $${\hat{d}}_{i}=\frac{\lambda_{i}}{1+\frac{N-1}{T}+\frac{2}{T}\sum_{j\neq i}\frac{\lambda_{j}}{\lambda_{i}-\lambda_{j}}}$$

Taking the inverse of Equation (10) yields:

(10) $${\hat{d}}_{i}^{-1}=\left(1-\frac{N-1}{T}\right)\lambda_{i}^{-1}+\left(\frac{N-1}{T}\right)\times 2\lambda_{i}^{-1}\hat{\theta }\left(\lambda_{i}^{-1}\right)\;$$

(11) $$\hat{\theta }\left(x\right)=\frac{1}{N-1}\overset{N}{\underset{\begin{array}{c} j=1\\ \begin{array}{c} \lambda_{j}^{-1}\neq x \end{array} \end{array}}{\sum }}\lambda_{j}^{-1}\frac{1}{\lambda_{j}^{-1}-x}\;\;\;\forall x\in R$$

In Equation (10), ${\hat{d}}_{i}^{-1}$ denotes the target estimator, where the first term represents the retained component and the second term represents the target smoothing component. $\hat{\theta }(x)$ serves as the weighted average of raw inverse eigenvalues. In Equation (11), $\lambda_{j}^{-1}/(\lambda_{j}^{-1}-x)$ measures the reciprocal of the difference between inverse eigenvalues, acting as a metric of “attractiveness” among eigenvalue estimates. This formulation clearly demonstrates linear shrinkage with respect to inverse eigenvalues, which combines $\lambda_{i}^{-1}$ and the smoothing term via a convex linear combination. The shrinkage intensity is (N−1)/T, allowing for stronger shrinkage as dimensionality increases.

The function $\hat{\theta }(x)$, referred to as the “Stein shrinkage,” possesses the following properties. First, it induces mutual attraction between eigenvalues. Second, higher-precision eigenvalues exert a stronger influence. Third, as the distance between eigenvalues increases, the denominator of the cross-term $\lambda_{j}^{-1}/(\lambda_{j}^{-1}-x)$ grows, leading the attractiveness measure to approach zero. Fourth, when eigenvalues are extremely close, the term approaches infinity.

The issue described in the fourth property above is addressed by the following novel smoothing formulation:

(12) $${\hat{d}}_{i}^{-1}=\left(1-\frac{N}{T}\right)\lambda_{i}^{-1}+\left(\frac{N}{T}\right)\times 2\lambda_{i}^{-1}\hat{\theta }\left(\lambda_{i}^{-1}\right)\;\;$$

(13) $$\hat{\theta }\left(x\right)=\frac{1}{N}\overset{N}{\underset{j=1}{\sum }}\lambda_{j}^{-1}\frac{\lambda_{j}^{-1}-x}{{\left(\lambda_{j}^{-1}-x\right)}^{2}+h^{2}\lambda_{j}^{-2}}\;$$

where the smoothing parameter $h∼KT^{-\alpha }$, with K>0, α∈(0,2/5). To address the issue of excessively large values caused by the fourth characteristic of the “Stein shrinkage [24]”, Equation (13) adopts the functional form to replace $1/(\lambda_{j}^{-1}-x)$ in Equation (11).

Based on Equation (13), this shrinkage quantity is referred to as the “smoothed Stein shrinkage”. When h=0, it is equivalent to Equation (11) and exhibits no smoothing effect; as h increases, the smoothing effect strengthens. If $\lambda_{i}^{-1}<\lambda_{j}^{-1}$, the influence of $\lambda_{j}^{-1}$ on $\lambda_{i}^{-1}$ is positive, indicating that ${\hat{d}}_{i}^{-1}$ tends to $\lambda_{j}^{-1}$; if $\lambda_{i}^{-1}>\lambda_{j}^{-1}$, the influence of $\lambda_{j}^{-1}$ on $\lambda_{i}^{-1}$ is negative, indicating that ${\hat{d}}_{i}^{-1}$ tends to decrease (i.e., tends to $\lambda_{j}^{-1}$). Consequently, this shrinkage exhibits locality: the impact of more distant eigenvalues decays rapidly as the distance increases.

The covariance matrix estimator is ultimately formulated as $\hat{\Sigma }=\sum_{i=1}^{N}{\hat{d}}_{i}\cdot u_{i}u_{i}^{\prime }$, which is referred to as “linear inverse shrinkage (LIS)”.

#### 2.3.2. Frobenius Loss Function

The Frobenius loss function is defined as:

(14) $$L^{FR}\left(\Sigma ,\hat{\Sigma }\right)=\frac{1}{N}Tr\left[{\left(\Sigma -\hat{\Sigma }\right)}^{2}\right]$$

Optimizing this loss function yields the following shrinkage estimator [24]:

(15) $${\hat{d}}_{i}^{-1}=\left(1-\frac{N}{T}\right)\lambda_{i}^{-1}+2\left(\frac{N}{T}\right)\left(1-\frac{N}{T}\right)2\lambda_{i}^{-1}\hat{\theta }\left(\lambda_{i}^{-1}\right)+{\left(\frac{N}{T}\right)}^{2}\lambda_{i}^{-1}A_{\hat{\theta }}^{2}\left(\lambda_{i}^{-1}\right)\;\;A_{\hat{\theta }}^{2}\left(x\right)={\left[\frac{1}{N}\overset{N}{\underset{j=1}{\sum }}\lambda_{j}^{-1}\frac{\lambda_{j}^{-1}-x}{{\left(\lambda_{j}^{-1}-x\right)}^{2}+h^{2}\lambda_{j}^{-2}}\right]}^{2}+\left[\frac{1}{N}\overset{N}{\underset{j=1}{\sum }}\lambda_{j}^{-1}\frac{h\lambda_{j}^{-1}}{{\left(\lambda_{j}^{-1}-x\right)}^{2}+h^{2}\lambda_{j}^{-2}}\right]$$

The conjugate of $\hat{\theta }\left(x\right)$ is given by:

(16) $${\hat{\theta }}^{*}(x)=\frac{1}{N}\sum_{j=1}^{N}\lambda_{j}^{-1}\frac{h\lambda_{j}^{-1}}{{\left(\lambda_{j}^{-1}-x\right)}^{2}+h^{2}\lambda_{j}^{-2}}$$

The first two terms of Equation (15) match the form of Equation (12) but differ in coefficients, with an additional third term. Following Ledoit and Péché [29], this third term constructs a quadratic oscillation term from $\hat{\theta }(x)$ and its conjugate ${\hat{\theta }}^{*}(x)$. The coefficients satisfy:

(17) $${\left(1-\frac{N}{T}\right)}^{2}+2\frac{N}{T}\left(1-\frac{N}{T}\right)+{\left(\frac{N}{T}\right)}^{2}={\left(1-\frac{N}{T}+\frac{N}{T}\right)}^{2}=1$$

Since the three weighting coefficients are quadratic functions of N/T, their sum forms a perfect square, leading to the name “quadratic inverse shrinkage (QIS)” for Equation (15).

#### 2.3.3. Kullback–Leibler Loss Function

The Kullback–Leibler loss function is defined as:

(18) $$L^{SKL}\left(\Sigma ,\hat{\Sigma }\right)=\frac{1}{2N}Tr\left(\Sigma^{-1}\hat{\Sigma }+\Sigma {\hat{\Sigma }}^{-1}\right)-1$$

By solving the optimization problem associated with the loss function, the optimal shrinkage estimator [24] is derived in the following form:

(19) $${\hat{\Sigma }}^{GIS}=\sum_{i=1}^{N}\sqrt{{\hat{\Sigma }}^{LIS}\times {\hat{\Sigma }}^{QIS}}\mu_{i}\mu_{i}^{T}$$

where ${\hat{\Sigma }}^{LIS}$ and ${\hat{\Sigma }}^{QIS}$ denote covariance matrix estimators computed via Equations (7) and (14), respectively. ${\hat{\Sigma }}^{GIS}$ is termed the “geometric inverse shrinkage (GIS)” estimator, obtained as the geometric mean of the ${\hat{\Sigma }}^{LIS}$ and ${\hat{\Sigma }}^{QIS}$ estimators.

Nonlinear shrinkage improves estimation by locally adjusting eigenvalues, thereby reducing noise and uncertainty in high-dimensional settings. Unlike global shrinkage methods, it adapts to the local structure of eigenvalue distributions, stabilizing higher-order moment estimation in complex systems where interactions among assets are nonlinear and highly interdependent.

### 2.4. Nonlinear Shrinkage Higher-Order Moment Estimation Process

The proposed nonlinear shrinkage estimation of higher-order moments proceeds as follows:

(1) Regress the return matrix on the factor model.

Using the factor model in Equation (4), estimates of $\hat{B}$ and $\hat{\varepsilon }$ are obtained.

(2) Estimate higher-order moments of residuals.

First, compute the eigenvalues and eigenvectors of the covariance matrix of residuals. Then, apply nonlinear shrinkage to the eigenvalues using the procedures in Equations (12), (15), and (19). Reconstruct the covariance matrix by combining the shrunken eigenvalues with the original eigenvectors, thereby obtaining the nonlinear shrinkage estimate ${\hat{\Delta }}_{\varepsilon }$. Substitute the variance elements of ${\hat{\Delta }}_{\varepsilon }$ into Equation (6) to derive the fourth-moment estimate ${\hat{\gamma }}_{\varepsilon }$. Additionally, define $\Omega_{\varepsilon ,ij}=E[e_{i}^{3}]$ to estimate the third-order moments of residuals.

(3) Estimate higher-order moments of factors.

Substitute factor data into Equation (1) to compute the $S_{F}$, $G_{F}$, and $P_{F}$.

(4) Aggregate moments of returns.

Substitute the residual multi-order moment estimates from Step (2) and the factor multi-order moment estimates from Step (3) into Equation (5) to derive the joint multi-order moment estimates of returns under nonlinear shrinkage.

## 3. Asymptotic Properties of Nonlinear Shrinkage Estimators

### 3.1. Consistency of Residual Higher-Order Moments

#### 3.1.1. Consistency of Second Moment

(1) Consistency of LIS

Ledoit and Wolf’s Theorem 3.1 shows that [23], under Assumptions 1–3, as both the sample size N and time dimension T tend to infinity, the covariance matrix estimator ${\hat{\Sigma }}^{LIS}$ yields a Stein loss function that converges in probability to the following deterministic limit:

(20) ∑k=1κ∫akbk1−c−2cxRem∨Fxxd^x−logd^xdFx+∫−∞+∞logtdHt−1

Thus, for all x∈Supp(F), we have $plim{\hat{\Sigma }}^{LIS}=\Sigma$, establishing the consistency of LIS.

(2) Consistency of QIS

Theorem 4.2 in Ledoit and Wolf [23] shows that, under Assumptions 1–3, as N and T tend to infinity, the ${\hat{\Sigma }}^{QIS}$ causes the Frobenius loss function to converge in probability to the following non-random limit:

(21) $$\int_{-\infty }^{+\infty }x^{2}dH\left(x\right)+\overset{\kappa }{\underset{k=1}{\sum }}\left\{-2\int_{a_{k}}^{b_{k}}\frac{x\hat{d}\left(x\right)}{{\left|1-c-cx{\overset{\vee }{m}}_{F}\left(x\right)\right|}^{2}}dF\left(x\right)+\int_{a_{k}}^{b_{k}}\hat{d}{\left(x\right)}^{2}dF\left(x\right)\right\}$$

Thus, for all x∈Supp(F), we have $plim\;\;{\hat{\Sigma }}^{QIS}=\Sigma$, establishing the consistency of QIS.

(3) Consistency of GIS

By Equations (20) and (21), the ${\hat{\Sigma }}^{GIS}$ in Equation (19) ensures that the Kullback–Leibler loss function converges in probability to the following nonrandom limit:

(22) 12∑k=1κ∫akbk1−c−2cxRem∨Fxxd^x+x1−c−cxmˇFx2d^xdFx−1

Thus, for all x∈Supp(F), we have $plim\;\;{\hat{\Sigma }}^{GIS}=\Sigma$, establishing the consistency of GIS.

To this point, the consistency of residual covariance estimators under the three loss functions has been demonstrated: $plim\;{\hat{\sigma }}_{\varepsilon }^{2}=\sigma_{\varepsilon }^{2}$. Detailed justifications for these consistency results can be found in Ledoit and Wolf [23].

#### 3.1.2. Consistency of Third Moment

The elements of ${\hat{\Omega }}_{\varepsilon }$ in the tensor are zero except for ${\hat{\Omega }}_{\varepsilon ,iii}$. Therefore, as N and T jointly tend to infinity, ${\hat{\Omega }}_{\varepsilon ,iii}$ converges to the following nonstochastic limit:

(23) $$\mathrm{plim}\;{\hat{\Omega }}_{\varepsilon ,iii}=plim\;\frac{1}{T}\sum_{t=1}^{T}{\left({\hat{\varepsilon }}_{it}-\mu_{\hat{\varepsilon },i}\right)}^{3}=E\left[{\left(\varepsilon_{i}-\mu_{\varepsilon ,i}\right)}^{3}\right]$$

where $\mu_{\hat{\varepsilon },i}$ denotes the residual sample mean vector for asset i. The expression $\sum_{t=1}^{T}{\left({\hat{\varepsilon }}_{it}-\mu_{\hat{\varepsilon },i}\right)}^{3}/T$ represents the sample estimate of residual coskewness in tensor form, which serves as a consistent estimator of $\Omega_{\varepsilon ,iii}$. Thus, $\mathrm{plim}\;{\hat{\Omega }}_{\varepsilon }=\Omega_{\varepsilon }$, establishing the consistency of the third-moment residual estimator.

#### 3.1.3. Consistency of Fourth Moment

The factor model is estimated using ordinary least squares (OLS). Under the standard OLS assumptions, $\hat{B}$ is asymptotically consistent, and ${\hat{\sigma }}_{F,q}^{2}$ serves as a consistent estimator of $\sigma_{F,q}^{2}$. The consistency of the ${\hat{\sigma }}_{\varepsilon }^{2}$ is proven in Equations (20)–(22). Since multiplication and addition in Equation (6) preserve consistency, the probability limit theorem implies that:

(24) $$plim\;{\hat{\gamma }}_{\varepsilon ,iiii}=plim\;\left(6\sum_{q=1}^{Q}{\hat{B}}_{iq}^{2}{\hat{\sigma }}_{F,q}^{2}{\hat{\sigma }}_{\varepsilon ,i}^{2}+E\left[{\hat{\varepsilon }}_{i}^{4}\right]\right)=6\sum_{q=1}^{Q}B_{iq}^{2}\sigma_{F,q}^{2}\sigma_{\varepsilon ,i}^{2}+E\left[\varepsilon_{i}^{4}\right]\;plim\;{\hat{\gamma }}_{\varepsilon ,iiil}=plim3\sum_{q=1}^{Q}{\hat{B}}_{iq}{\hat{B}}_{lq}{\hat{\sigma }}_{F,q}^{2}{\hat{\sigma }}_{\varepsilon ,i}^{2}=3\sum_{q=1}^{Q}B_{iq}B_{lq}\sigma_{F,q}^{2}\sigma_{\varepsilon ,i}^{2}\;\begin{array}{c} plim{\hat{\gamma }}_{\varepsilon ,iikk}=plim\;\left(\sum_{q=1}^{Q}{\hat{B}}_{iq}^{2}{\hat{\sigma }}_{F,q}^{2}{\hat{\sigma }}_{\varepsilon ,i}^{2}+\sum_{q=1}^{Q}{\hat{B}}_{lq}^{2}{\hat{\sigma }}_{F,q}^{2}{\hat{\sigma }}_{\varepsilon ,i}^{2}+{\hat{\sigma }}_{\varepsilon ,i}^{2}{\hat{\sigma }}_{\varepsilon ,l}^{2}\right)\\ =\sum_{q=1}^{Q}B_{iq}^{2}\sigma_{F,q}^{2}\sigma_{\varepsilon ,i}^{2}+\sum_{q=1}^{Q}B_{lq}^{2}\sigma_{F,q}^{2}\sigma_{\varepsilon ,i}^{2}+\sigma_{\varepsilon ,i}^{2}\sigma_{\varepsilon ,l}^{2} \end{array}\;plim\;{\hat{\gamma }}_{\varepsilon ,iikl}=plim\;\sum_{q=1}^{Q}{\hat{B}}_{kq}{\hat{B}}_{lq}{\hat{\sigma }}_{F,q}^{2}{\hat{\sigma }}_{\varepsilon ,i}^{2}=\sum_{q=1}^{Q}B_{kq}B_{lq}\sigma_{F,q}^{2}\sigma_{\varepsilon ,i}^{2}$$

Therefore, when N and T tend to infinity jointly, $plim\;{\hat{\gamma }}_{\varepsilon }=\gamma_{\varepsilon }$, establishing the consistency of the fourth-moment estimator for residuals.

### 3.2. Consistency of Returns Higher-Order Moments

Assume that Assumptions 1–3 of Ledoit and Wolf [23] hold, along with the basic assumptions of OLS. When N and T tend to infinity jointly, the higher-order moments of returns covariances converge to the following nonstochastic limits:

(25) $$\mathrm{plim}\hat{V}=BS_{F}B^{T}+\Delta_{\varepsilon }\;plim\;\hat{S}\;=BG_{F}\left(B^{T}⊗B^{T}\right)+\Omega_{\varepsilon }\;\mathrm{plim}\;\hat{K}=BP_{F}\left(B^{T}⊗B^{T}⊗B^{T}\right)+\gamma_{\varepsilon }$$

**Proof.**  ${\hat{S}}_{F}$, ${\hat{G}}_{F}$ and ${\hat{P}}_{F}$ denote the second-moment, third-moment, and fourth-moment estimators of the factor sample data. When N and T tend to infinity jointly, their elements converge to the following nonstochastic limits:

(26) $$\;plim\;{\hat{\upsilon }}_{F,om}=plim\frac{1}{T}\sum_{t=1}^{T}\left({\hat{F}}_{o}-{\hat{\mu }}_{F,o}\right)\left({\hat{F}}_{m}-{\hat{\mu }}_{F,m}\right)=\;E\left[\left(F_{o}-\mu_{F,o}\right)\left(F_{m}-\mu_{F,m}\right)\right]\;\begin{array}{c} plim\;{\hat{s}}_{F,omg}=plim\frac{1}{T}\;\sum_{t=1}^{T}\left({\hat{F}}_{o}-{\hat{\mu }}_{F,o}\right)\left({\hat{F}}_{m}-{\hat{\mu }}_{F,m}\right)\left({\hat{F}}_{g}-{\hat{\mu }}_{F,g}\right)\\ =\;E\left[\left(F_{o}-\mu_{F,o}\right)\left(F_{m}-\mu_{F,m}\right)\left(F_{g}-\mu_{F,g}\right)\right] \end{array}\;\begin{array}{c} plim\;{\hat{k}}_{F,omgc}=plim\frac{1}{T}\sum_{t=1}^{T}\left({\hat{F}}_{o}-{\hat{\mu }}_{F,o}\right)\left({\hat{F}}_{m}-{\hat{\mu }}_{F,m}\right)\left({\hat{F}}_{g}-{\hat{\mu }}_{F,g}\right)\left({\hat{F}}_{c}-{\hat{\mu }}_{F,c}\right)\;\\ =E\left[\left(F_{o}-\mu_{F,o}\right)\left(F_{m}-\mu_{F,m}\right)\left(F_{g}-\mu_{F,g}\right)\left(F_{c}-\mu_{F,c}\right)\right] \end{array}$$

where the quantities ${\hat{\upsilon }}_{F,om}$, ${\hat{s}}_{F,omg}$, and ${\hat{k}}_{F,omgc}$ are, respectively, mapped to elements of ${\hat{S}}_{F}$, ${\hat{G}}_{F}$, and ${\hat{P}}_{F}$ for all o,m,g,c∈1,⋯,Q. Hence, the consistency of ${\hat{S}}_{F}$, ${\hat{G}}_{F}$, and ${\hat{P}}_{F}$ is established. By combining the consistency of the factor and residual moments with that of $\hat{B}$, the estimator of the higher-order moments of returns converges to the following limit:

(27) $$\begin{array}{c} plim\;\hat{V}=plim\;\hat{B}{\hat{S}}_{F}{\hat{B}}^{T}+plim\;{\hat{\Delta }}_{\varepsilon }=BS_{F}B^{T}+\Delta_{\varepsilon }\\ plim\;\hat{S}=plim\;\hat{B}{\hat{G}}_{F}\left({\hat{B}}^{T}⊗{\hat{B}}^{T}\right)+plim\;{\hat{\Omega }}_{\varepsilon }=BG_{F}\left(B^{T}⊗B^{T}\right)+\Omega_{\varepsilon }\\ plim\;\hat{K}=\mathrm{plim}\;\hat{B}{\hat{P}}_{F}\left({\hat{B}}^{T}⊗{\hat{B}}^{T}⊗{\hat{B}}^{T}\right)+\mathrm{plim}\;{\hat{\gamma }}_{\varepsilon }=BP_{F}\left(B^{T}⊗B^{T}⊗B^{T}\right)+\gamma_{\varepsilon } \end{array}$$

To this point, the consistency of the nonlinear shrinkage estimator of returns’ higher-order moments under the large-sample framework has been proven. □

## 4. Monte Carlo Simulation

### 4.1. Simulation Design

We use constituent stocks of China’s A-share market and obtain weekly return data from the Wind database, spanning January 2006 to December 2020. Parameters of factor loadings and residual structures in the multifactor models are estimated using OLS and subsequently employed to construct the data-generating process (DGP) for the Monte Carlo simulation design. A 2 × 3 sorting method is applied to construct multifactor investment portfolios. The five-factor model includes the market excess return (MKT-RF), the size factor SMB (Small Minus Big), the value factor HML (High Minus Low), the profitability factor RMW (Robust Minus Weak), and the investment factor CMA (Conservative Minus Aggressive). The three-factor model retains MKT-RF, SMB, and HML. In the Monte Carlo simulations, asset dimensionality is set to 5, 10, and 30, while sample sizes are set to 50, 100, 500, and 1000. By varying the number of assets and sample sizes, we evaluate how nonlinear shrinkage estimation manages uncertainty across different levels of system complexity. For each parameter configuration, 200 independent replications are performed to ensure the robustness and representativeness of the statistical results. The Monte Carlo simulation proceeds as follows:

(1) Generate factor data F

We first generate the distributional parameters of the factor data, including the location vector ${\hat{u}}_{F}$, scale matrix ${\hat{\Omega }}_{F}$, skewness parameters ${\hat{\alpha }}_{F}$, and degrees of freedom ${\hat{\nu }}_{F}$, based on the underlying factor distribution. We then simulate a random sample of factor data with sample size T from the multivariate skew-t distribution $ST({\hat{u}}_{F},{\hat{\Omega }}_{F},{\hat{\alpha }}_{F},{\hat{\nu }}_{F})$.

(2) Generate factor loadings B

The distribution parameters of the factor loading matrix are estimated using OLS, yielding ${\hat{u}}_{B},{\hat{\Omega }}_{B},{\hat{\alpha }}_{B},$ and ${\hat{\nu }}_{B}$. A random sample of the factor loading matrix is then simulated from the multivariate skew-t distribution $ST({\hat{u}}_{B},{\hat{\Omega }}_{B},{\hat{\alpha }}_{B},{\hat{\nu }}_{B})$.

(3) Generate residuals ε

The probability density function of ε is specified in Equation (28), where ν denotes the degrees of freedom, ξ is the asymmetry parameter, and m and s represent the location and scale parameters of the skewed Student’s t distribution, respectively. Residuals are initially obtained from OLS. Based on these estimates, the parameter vector $\left(\hat{m},\hat{s},\hat{\nu },\hat{\xi }\right)$ is obtained under the skewed Student’s t distribution, and subsequently used to simulate the residual vector ε.

(28) $$f\left(\varepsilon |s,\nu \right)=\left\{\begin{array}{c} -\frac{2}{\xi +\frac{1}{\xi }}sg\left[\xi \left(s\varepsilon +m\right)|\nu \right],\varepsilon <-\frac{m}{s}\\ -\frac{2}{\xi +\frac{1}{\xi }}sg\left[\frac{\left(s\varepsilon +m\right)}{\xi }|\nu \right],\varepsilon \geq -\frac{m}{s} \end{array}\right.$$

(4) Generate returns R

Using the outputs from Steps (1)–(3), we substitute them into Equation (4) to obtain a random sample of returns of length  T .

(5) Compute the PRIAL

We first compute the MSE of the sample estimator, the linear shrinkage estimator (SN), and the nonlinear shrinkage estimator (SH) under three loss functions. Using these MSEs, we then calculate PRIAL values of the nonlinear shrinkage estimator relative to both the sample and linear shrinkage estimators. PRIAL is given by

(29) $$\begin{array}{c} PRIAL=\left(\frac{S-SH}{S}\right)\times 100\%\\ PRIAL=\left(\frac{SN-SH}{SN}\right)\times 100\% \end{array}$$

Higher PRIAL values indicate that the nonlinear shrinkage estimator achieves smaller MSE compared with the sample and linear shrinkage estimators, thus demonstrating the superior accuracy of the proposed method.

### 4.2. Simulation Results

Table 2 reports PRIAL values for higher-order moment matrices, computed from return data generated by the five-factor model. Positive PRIAL values indicate not only that the MSE of the nonlinear shrinkage estimator is smaller than that of the sample and linear shrinkage estimators, but also that the associated estimation uncertainty is relatively lower. These results demonstrate that the nonlinear shrinkage estimator for higher-order moment matrices enhances the precision of estimating covariance, coskewness, and cokurtosis matrices. Similarly, Table A1 and Table A2 also exhibit similar results in Appendix A.1.

When applying nonlinear shrinkage estimation to covariance matrices, PRIAL results indicate that this approach outperforms both the sample and linear shrinkage methods. For a fixed number of observations T, PRIAL values gradually increase as the number of assets N grows. For instance, when T=1000, as N increases from 5 to 30, the PRIAL value of SHFF rises from 12.943 to 35.023, and that of FF rises from 11.683 to 31.137. The results show that as the number of assets increases, nonlinear shrinkage more effectively stabilizes estimation in high-dimensional complex systems, filtering out noise while preserving meaningful dependence structures. Conversely, for a fixed number of assets N, PRIAL values generally decline as the number of observations T increases. For example, when N=5, as T increases from 50 to 1000, the PRIAL value of SHFF decreases from 19.623 to 12.943, and that of FF decreases from 18.331 to 11.683. This indicates that as the data quantity grows, the relative improvement offered by nonlinear shrinkage methods narrows, although their absolute precision remains superior to traditional approaches. Overall, the improvement effect becomes more pronounced with increasing N and diminishes with increasing T.

In the context of coskewness matrix estimation, PRIAL results indicate that nonlinear shrinkage estimation, compared to sample estimation, leads to a gradual increase in the SHFF value as the number of assets N rises while the number of observations T is held constant. For example, when T=1000, the PRIAL value increases from 78.276 to 91.644 as N grows from 5 to 30. This finding indicates that nonlinear shrinkage methods can more accurately capture asymmetric dependence structures among asset returns when the investment portfolio includes a larger number of assets. Such improvement enables investors to identify assets with positive skewness (i.e., right-skewed return distributions) and optimize portfolio allocations to exploit asymmetric return opportunities. When the number of assets N is fixed and the number of observations T increases, the PRIAL value of the SHFF also shows a gradual upward trend. For example, when N=5, as T increases from 50 to 1000, the PRIAL value rises from 26.229 to 78.276. This demonstrates that nonlinear shrinkage estimation can more effectively filter out noise in coskewness data as the data volume expands, facilitating investors’ allocation of assets with positive skewness to achieve higher returns. Overall, the enhancement effects intensify with increases in both the number of assets N and the number of observations T. Although the PRIAL value of the FF specification is positive, it is much smaller, indicating that the estimation performances of the two approaches are very similar. This outcome is primarily driven by the structural design of the proposed method.

For cokurtosis matrix estimation, the nonlinear shrinkage estimator outperforms both the sample and linear shrinkage estimators. When the observations T is fixed, PRIAL values gradually rise as the number of assets N increases. For example, when T=1000 and N increases from 5 to 30, the SHFF-based PRIAL value rises from 66.977 to 89.219. This pattern indicates that in high-dimensional portfolio settings, the nonlinear shrinkage estimator improves accuracy by locally adjusting the eigenvalue distribution of the cokurtosis matrix. The resulting gains help investors identify tail co-movement and crash-prone configurations, thereby strengthening tail-risk management. When the number of assets N is fixed, increasing the observations T leads to a gradual rise in the PRIAL value. For example, with N=5, as T increases from 50 to 1000, the PRIAL value of SHFF increases from 26.145 to 66.977. Larger samples enable the nonlinear shrinkage estimator to significantly mitigate estimation bias in the cokurtosis matrix, allowing investors to more reliably extract tail-dependence structures from historical data. By comparison, the PRIAL values for FF remain consistently positive, confirming the superior precision of nonlinear shrinkage methods, although no systematic directional trend is observed.

These improvements reflect not only better risk–return trade-offs but also enhanced resilience to systemic uncertainty. Table A3, Table A4 and Table A5 report the PRIAL values of higher-order moments based on return data generated by the three-factor model. The results show that the nonlinear shrinkage estimator continues to improve the estimation accuracy of higher-order moments, consistent with the findings under the five-factor model.

## 5. Empirical Analysis

### 5.1. Data Processing

This paper employs weekly returns of constituent stocks in China’s A-share market from January 2006 to December 2020. To ensure sample consistency and reliability, stocks with trading suspensions exceeding 20 consecutive weeks during the sample period are excluded. After screening, 100 stocks are retained, yielding 764 usable weekly observations. Subsequently, subsamples of different sizes (e.g., 10 stocks, 30 stocks) are randomly selected for analysis. For sample partitioning, data from January 2006 to December 2010 are used as the training set to estimate model parameters, while data from January 2011 to December 2020 constitute the test set for model validation and portfolio evaluation. To enhance estimation timeliness and adaptability in the presence of uncertainty, a rolling-window estimation procedure is employed, which allows the model to adjust continuously to the evolving dynamics of complex financial systems. In each period, the higher-order moment matrices are estimated using the preceding five years of historical data and applied to portfolio construction.

### 5.2. Maximizing Expected Utility Portfolio

The portfolio objective function is constructed under a CRRA preference framework, incorporating variance, skewness, and kurtosis. We compare portfolios estimated via nonlinear shrinkage with those based on sample and linear shrinkage estimators to identify optimal asset allocations. This comparison highlights each method’s ability to manage estimation uncertainty and systemic complexity in portfolio construction. Assuming zero expected returns for all assets, the portfolio objective function with short-sale constraints is given by:

(30) $$\begin{array}{c} \underset{\omega }{\max}\left\{-\frac{\tau }{2}\omega^{T}\hat{V}\omega +\frac{\tau (\tau +1)}{6}\omega^{T}\hat{S}\left(\omega ⊗\omega \right)-\frac{\tau (\tau +1)(\tau +2)}{24}\omega^{T}\hat{K}\left(\omega ⊗\omega ⊗\omega \right)\right\}\\ st.\;:\;\omega^{T}1_{n}=1\\ \omega_{i}\geq 0,i=1,\cdots ,N \end{array}$$

where $\omega_{i}$ denotes the weight of stock i, and τ represents the risk-aversion coefficient. This paper considers two levels of risk aversion, τ=5 and τ=10. To evaluate the practical applicability of the proposed methodology, we conduct out-of-sample performance tests on portfolios constructed under different estimation frameworks. In addition to annualized return (AR), we assess portfolio performance using multiple metrics, including annualized volatility (AV), value at risk (VaR), Sharpe ratio (SR), kurtosis, and maximum drawdown (MD), to provide a systematic evaluation from both return and risk perspectives.

Table 3 reports the out-of-sample portfolio performance of various estimation methods when the number of assets is 30 and the number of factors is 5. Results from the fourth-order approximation of the investor utility function are reported, where the risk-aversion coefficient is set to 5. The results reveal the following:

(1) In terms of annualized return, the portfolio constructed using the nonlinear shrinkage estimator outperforms all other methods, followed by the five-factor approach; both substantially exceed the linear shrinkage and sample covariance estimators. This indicates that nonlinear shrinkage estimation more effectively captures return structures, thereby improving portfolio performance.
(2) Regarding the Sharpe ratio, the nonlinear shrinkage method again yields the highest risk-adjusted return, demonstrating its ability to generate excess returns while controlling risk. The five-factor model ranks second, whereas the sample covariance and linear shrinkage methods perform relatively weakly. This suggests that the nonlinear shrinkage portfolio achieves higher excess returns per unit of risk, thereby combining high annualized return with superior downside protection.
(3) With respect to the maximum drawdown ratio, the nonlinear shrinkage estimator slightly outperforms other methods in extreme risk control, producing the smallest drawdown magnitude. A lower maximum drawdown ratio implies that this estimator more effectively mitigates potential losses under extreme market conditions, enhancing portfolio resilience to tail risk.
(4) Based on the fourth-order CRRA utility expansion, nonlinear shrinkage estimators yield the highest gains in expected utility, indicating their superior risk-adjusted performance. For moderate risk aversion (τ=5), the utility gains range between 4 and 5%, while for more risk-averse investors (τ=10), the improvements increase to approximately 6–8%. These gains are mainly driven by lower kurtosis and reduced tail risk.

**Table 3:** Out-of-sample portfolio performance under CRRA utility with 30 assets and five factors across different methods.

Coefficient	Method	AR	AV	VaR	SR	Kurtosis	MD	Utility Gain
τ=5	Sample	11.837	0.216	−0.241	0.548	4.259	22.103	-
	Five-Factor	13.033	0.221	−0.238	0.593	3.980	20.379	3.65%
	Linear	11.667	0.212	−0.232	0.549	4.201	20.925	4.12%
	QIS	13.439	0.220	−0.238	0.609	3.880	20.212	5.26%
	LIS	13.187	0.222	−0.242	0.594	3.911	20.156	4.67%
	GIS	13.354	0.221	−0.240	0.603	3.896	20.225	5.25%
τ=10	Sample	11.709	0.217	−0.244	0.539	4.232	22.407	-
	Five-Factor	12.666	0.222	−0.241	0.576	4.019	21.190	4.85%
	Linear	11.379	0.213	−0.234	0.535	4.199	21.273	4.27%
	QIS	13.090	0.221	−0.241	0.592	3.917	20.993	8.13%
	LIS	12.828	0.222	−0.244	0.577	3.945	21.028	6.18%
	GIS	13.000	0.221	−0.242	0.587	3.930	21.153	6.45%

These results underscore the capacity of nonlinear shrinkage to reduce uncertainty and strengthen portfolio robustness. While measures such as VaR and annualized volatility display relatively similar outcomes across methods, the kurtosis of the nonlinear shrinkage estimator remains the lowest. This highlights its superior performance in mitigating extreme values in the return distribution and reducing exposure to large market fluctuations. Furthermore, the nonlinear shrinkage estimators enhance investors’ welfare by delivering more stable risk-adjusted performance. A 4–8% improvement in CRRA utility corresponds to a substantial increase in certainty-equivalent wealth, which can offset moderate transaction costs or management fees typically observed in institutional portfolios. In addition, the reduction in higher-order risks (particularly kurtosis) suggests that the proposed estimators are especially valuable in large-asset settings, where diversification alone cannot fully eliminate tail dependencies. Overall, under comparable levels of annualized volatility and maximum drawdown, the nonlinear shrinkage portfolio not only delivers higher annualized returns but also achieves a higher Sharpe ratio. This implies that investors can obtain greater returns without taking on additional risk, or bear lower risk for the same expected return level—an outcome particularly relevant for those pursuing high risk-adjusted performance.

When the risk aversion parameter τ increases from 5 to 10, it can be observed that both the average annualized return and the Sharpe ratio generally decline, though the magnitude of this decline is not substantial. Meanwhile, other metrics remain largely unchanged. This suggests that nonlinear shrinkage estimation still exhibits favorable properties.

Table 4 reports the out-of-sample portfolio performance of different estimation methods when the number of assets is small (N=10). The results reveal the following:

(1) Annualized returns are relatively similar across methods, with sample estimates delivering the highest returns and the five-factor model delivering the lowest. Nonlinear and linear shrinkage show only minor differences in return prediction, suggesting comparable performance in capturing return dynamics.
(2) The ranking of Sharpe ratios closely mirrors that of annualized returns. Nonlinear shrinkage performs slightly worse than linear shrinkage, indicating that when portfolios consist of a small number of assets, simple linear methods may sufficiently capture the key risk–return trade-offs. In such cases, linear shrinkage offers comparable effectiveness while being more parsimonious and operationally efficient.
(3) Analysis of kurtosis shows that nonlinear shrinkage yields lower kurtosis than linear shrinkage, implying better mitigation of extreme values, a more concentrated return distribution, and improved portfolio stability.
(4) Comparing Table 4 with Table 3 reveals that as the asset dimension increases from 10 to 30, the advantages of nonlinear shrinkage in annualized return, Sharpe ratio, and kurtosis become more pronounced. Although higher dimensionality enhances diversification and thus mean returns, it also introduces greater estimation uncertainty and tail risk, leading to slightly smaller CRRA utility gains. This outcome reflects a rational trade-off between improved returns and controlled risk exposure. Even so, under lower risk aversion (τ=5), nonlinear shrinkage continues to outperform the sample and linear estimators, indicating its effectiveness in capturing complex interdependencies among a larger set of assets and enhancing overall portfolio robustness.

**Table 4:** Out-of-sample portfolio performance under CRRA utility with 10 assets and five factors across different methods.

Coefficient	Method	AR	AV	VaR	SR	Kurtosis	MD	Utility Gain
τ=5	Sample	12.167	0.202	−0.223	0.604	4.611	18.666	-
	Five-Factor	10.958	0.194	−0.217	0.564	4.356	18.218	6.43%
	Linear	11.545	0.198	−0.220	0.582	4.389	18.191	4.06%
	QIS	11.430	0.196	−0.217	0.582	4.264	18.095	5.32%
	LIS	11.305	0.196	−0.217	0.578	4.274	18.122	5.27%
	GIS	11.379	0.196	−0.217	0.581	4.269	18.025	5.35%
τ=10	Sample	11.958	0.202	−0.228	0.593	4.785	19.218	-
	Five-Factor	10.334	0.191	−0.217	0.540	4.464	18.342	8.94%
	Linear	10.989	0.196	−0.221	0.560	4.510	18.257	6.46%
	QIS	10.832	0.193	−0.217	0.553	4.369	18.112	10.12%
	LIS	10.684	0.193	−0.217	0.554	4.383	18.138	9.95%
	GIS	10.772	0.193	−0.217	0.557	4.376	18.126	10.02%

Table 5 further compares the out-of-sample forecasting performance of the different estimators. The results indicate that, across all moment orders, the nonlinear shrinkage method outperforms alternative approaches, confirming its effectiveness in enhancing estimation accuracy. Moreover, the PRIAL values of the nonlinear shrinkage estimator relative to the linear shrinkage estimator are overall positive, with the most pronounced improvements observed for covariance and cokurtosis, while the gains for coskewness are relatively limited. These findings are consistent with the conclusions drawn from the preceding simulation analysis.

In summary, by optimizing the eigenvalue spectrum, nonlinear shrinkage improves both estimation accuracy and robustness, representing a practical approach for portfolio construction.

## 6. Robustness Checks

We next conduct robustness checks on the preceding results. Specifically, we vary the number of factors and the number of assets to re-examine the conclusions using the proposed nonlinear shrinkage estimator for covariance and higher-order moments.

Table 6 presents the out-of-sample portfolio performance of different methods with 30 assets when the number of factors is reduced to three. The results show that the performance under the three-factor model is comparable to that under the five-factor model. This suggests that even with fewer factors, nonlinear estimation methods can still deliver robust returns, highlighting their adaptability and reliability. More importantly, the robustness across factor specifications illustrates the method’s ability to manage estimation uncertainty in complex financial systems.

Table 7 presents the out-of-sample forecasting performance with 30 assets when the number of factors is reduced to three. The results show that the nonlinear shrinkage estimator achieves higher predictive accuracy than both the linear shrinkage and sample estimators, confirming its robustness within a three-factor framework. From an economic perspective, the nonlinear shrinkage estimator sustains relatively high PRIAL values even with fewer factors, highlighting its adaptability and stability in capturing higher-order dependence structures. These findings confirm that the method remains effective under different system complexities, reducing uncertainty even when factor information is limited.

Table 8 reports the out-of-sample forecasting performance with five factors when the number of assets increases to ten. Consistent with the previous analysis, the results emphasize the stability of the proposed approach across different model specifications.

## 7. Conclusions

This paper extends nonlinear shrinkage estimation from covariance matrices to higher-order moment matrices within a multifactor framework. By integrating factor-model dimension reduction, tensor supersymmetry, and nonlinear eigenvalue shrinkage, the proposed approach mitigates dimensionality issues and enhances estimation robustness in complex financial systems. Theoretical results establish its asymptotic consistency, and Monte Carlo simulations confirm substantial reductions in MSE and improvements in PRIAL relative to sample and linear shrinkage benchmarks. Empirical evidence from weekly A-share data demonstrates that portfolios constructed from nonlinear-shrinkage higher-order moments achieve higher risk-adjusted performance, characterized by increased annualized returns, reduced kurtosis, and smaller drawdowns, particularly in large-asset universes. These findings highlight the practical economic relevance of directly addressing estimation uncertainty through localized eigenvalue adjustment.

This study also contributes to the broader literature on random matrix theory, robust covariance estimation, and high-dimensional portfolio optimization. The concept of shrinkage estimation originated from Stein’s pioneering work on multi-parameter estimation [28]. Building on this foundation, Ledoit and Wolf and Ledoit and Péché developed eigenvalue-shrinkage techniques under the random matrix theory framework [29,30,31], establishing a rigorous foundation for noise reduction in large-dimensional covariance estimation. Subsequent advances in robust and approximate factor modeling by Bai and Ng, Fan, Liao, and Mincheva, and Onatski further addressed weak-factor structures and cross-sectional dependence [25,26,27]. From an asset-pricing perspective, stochastic volatility and multifactor models [32,33] link higher-order moment dynamics to option-implied risk premia [32,34]. Future research could explore dynamic extensions such as time-varying shrinkage [33], dynamic equicorrelation [35], regime-switching dependence [36], and realized high-frequency moment estimation [37] to better capture market nonstationarity and structural change.

## Figures and Tables

**Table 1:** Dimensionality reduction in higher-order moment tensors under supersymmetry.

Tensor Order	Without Supersymmetry	With Supersymmetry	Dimension Reduction
Covariance	$N^{2}$	$\frac{N(N+1)}{2}$	Removes symmetric duplicates (i,j=j,i)
Coskewness	$N^{3}$	$\frac{N(N+1)(N+2)}{6}$	Exploits full permutation invariance (i,j,k)
Cokurtosis	$N^{4}$	$\frac{N(N+1)(N+2)(N+3)}{24}$	Reduces exponential growth to polynomial scale

**Table 2:** Improvements in the five-factor QIS over sample and linear shrinkage in higher-order moment estimation.

		Covariance		Coskewness		Cokurtosis	
N	T	SHFF *	FF	SHFF	FF	SHFF	FF
5	50	19.623	18.331	26.229	5.725	26.145	12.320
	100	34.038	32.302	58.954	5.605	69.302	45.722
	500	11.973	11.173	65.363	3.132	58.217	45.209
	1000	12.943	11.683	78.276	3.152	66.977	30.652
10	50	50.002	44.770	82.706	0.355	77.045	44.172
	100	53.020	48.502	88.677	1.596	85.795	60.894
	500	38.160	35.020	86.681	6.067	85.253	58.894
	1000	30.357	28.719	89.182	2.775	86.284	68.653
30	50	64.303	58.442	85.041	1.307	84.349	53.581
	100	59.716	53.443	90.000	0.441	90.254	64.685
	500	51.191	45.237	88.229	1.333	87.293	60.369
	1000	35.023	31.137	91.644	0.041	89.219	60.347

* SHFF denotes the PRIAL values of QIS compared to the sample estimator, and FF denotes the PRIAL values of QIS compared to linear shrinkage. Five-factor LIS and GIS are presented in Table A1 and Table A2, respectively; three-factor LIS, QIS, and GIS are presented in Table A3, Table A4 and Table A5, respectively.

**Table 5:** Out-of-sample moment improvements in nonlinear shrinkage relative to linear shrinkage and sample estimators with 30 assets and five factors.

Method	Moment	Mean	St. Dev.	Minimum	Maximum	Median
Linear and Sample	*V*	6.067	0.068	−1.626	17.789	5.129
	*S*	33.992	0.225	−0.606	64.609	40.689
	*K*	22.582	0.278	−24.651	69.494	20.988
QIS and Sample	*V*	8.414	0.081	−0.334	24.839	6.987
	*S*	33.992	0.225	−0.606	64.609	40.689
	*K*	23.087	0.282	−24.023	70.086	21.807
LIS and Sample	*V*	9.384	0.096	−1.623	29.419	8.551
	*S*	33.992	0.226	−0.606	64.609	40.689
	*K*	23.841	0.289	−23.591	71.234	23.491
GIS and Sample	*V*	8.537	0.083	−0.421	25.695	7.122
	*S*	33.992	0.226	−0.606	64.609	40.689
	*K*	23.186	0.283	−24.058	70.077	22.000
QIS and Linear	*V*	2.558	0.039	−5.304	8.576	2.504
	*S*	0.000	0.000	0.000	0.000	0.000
	*K*	0.950	0.019	−0.751	5.898	0.281
LIS and Linear	*V*	3.664	0.057	−5.689	14.147	2.508
	*S*	0.000	0.000	0.000	0.000	0.000
	*K*	2.390	0.039	−1.772	11.494	1.328
GIS and Linear	*V*	2.694	0.042	−5.624	9.616	2.466
	*S*	0.000	0.000	0.000	0.000	0.000
	*K*	1.110	0.022	−0.575	6.825	0.345

**Table 6:** Out-of-sample portfolio performance under CRRA utility with 30 assets and three factors across different methods.

Coefficient	Method	AR	AV	VaR	SR	Kurtosis	MD
τ=5	Sample	11.840	0.216	−0.241	0.548	4.260	22.146
	Three-Factor	12.901	0.218	−0.235	0.591	3.972	20.400
	Linear	12.686	0.214	−0.231	0.565	4.167	20.548
	QIS	13.255	0.220	−0.235	0.604	3.861	20.154
	LIS	13.000	0.221	−0.239	0.589	3.891	20.245
	GIS	13.169	0.220	−0.236	0.599	3.872	20.389
τ=10	Sample	11.712	0.217	−0.244	0.539	4.231	22.409
	Three-Factor	12.557	0.219	−0.238	0.574	4.008	20.709
	Linear	11.788	0.214	−0.235	0.550	4.196	20.883
	QIS	12.937	0.220	−0.238	0.588	3.894	20.552
	LIS	12.673	0.221	−0.242	0.573	3.921	20.948
	GIS	12.843	0.220	−0.239	0.583	3.905	20.694

**Table 7:** Out-of-sample moment improvements in nonlinear shrinkage relative to linear shrinkage and sample estimators with 30 assets and three factors.

Method	Moment	Mean	St. Dev.	Minimum	Maximum	Median
Linear and Sample	*V*	7.342	0.064	0.891	17.978	7.275
	*S*	33.329	0.217	−0.679	64.503	37.754
	*K*	23.656	0.242	−8.444	67.500	19.374
QIS and Sample	*V*	8.793	0.085	−0.870	24.932	8.099
	*S*	33.329	0.218	−0.679	64.503	37.754
	*K*	24.156	0.246	−7.804	68.173	20.133
LIS and Sample	*V*	9.803	0.098	−2.101	29.466	8.700
	*S*	33.329	0.218	−0.679	64.503	37.754
	*K*	24.921	0.253	−7.523	69.347	21.879
GIS and Sample	*V*	8.894	0.086	−0.874	25.597	8.195
	*S*	33.329	0.218	−0.679	64.503	37.754
	*K*	24.240	0.246	−7.819	68.102	20.326
QIS and Linear	*V*	1.700	0.031	−3.614	8.477	1.463
	*S*	0.000	0.000	0.000	0.000	0.000
	*K*	0.911	0.018	−0.862	5.607	0.322
LIS and Linear	*V*	2.869	0.050	−4.035	14.005	2.747
	*S*	0.000	0.000	0.000	0.000	0.000
	*K*	2.313	0.037	−1.805	10.703	1.308
GIS and Linear	*V*	1.811	0.034	−4.054	9.288	1.440
	*S*	0.000	0.000	0.000	0.000	0.000
	*K*	1.029	0.020	−0.616	6.233	0.320

**Table 8:** Out-of-sample moment improvements in nonlinear shrinkage relative to linear shrinkage and sample estimators with 10 assets and five factors.

Method	Moment	Mean	St. Dev.	Minimum	Maximum	Median
Linear and Sample	*V*	2.441	0.069	−5.261	18.708	0.510
	*S*	33.654	0.320	−2.722	87.155	33.350
	*K*	17.556	0.423	−64.841	81.254	14.497
QIS and Sample	*V*	6.463	0.097	−8.101	20.848	8.868
	*S*	33.654	0.320	−2.722	87.155	33.350
	*K*	18.444	0.428	−65.122	81.397	15.460
LIS and Sample	*V*	7.671	0.107	−8.997	22.599	9.725
	*S*	33.654	0.320	−2.722	87.155	33.350
	*K*	19.305	0.430	−64.409	81.734	17.656
GIS and Sample	*V*	6.638	0.098	−8.090	21.200	9.075
	*S*	33.654	0.320	−2.722	87.155	33.350
	*K*	18.631	0.428	−64.978	81.382	15.901
QIS and Linear	*V*	4.179	0.060	−4.570	11.495	1.945
	*S*	0.000	0.000	0.000	0.000	0.000
	*K*	1.598	0.025	−1.566	7.318	1.155
LIS and Linear	*V*	5.453	0.071	−5.437	14.790	4.570
	*S*	0.000	0.000	0.000	0.000	0.000
	*K*	2.992	0.033	−1.690	10.083	2.800
GIS and Linear	*V*	4.364	0.061	−4.560	12.274	2.479
	*S*	0.000	0.000	0.000	0.000	0.000
	*K*	1.828	0.026	−1.571	7.817	1.551

## Data Availability

The data and code are available in the figshare repository at https://doi.org/10.6084/m9.figshare.30179059.

## Appendix A

### Appendix A.1

**Table A1:** Improvements in the five-factor LIS over sample and linear shrinkage in higher-order moment estimation.

		Covariance		Coskewness		Cokurtosis	
N	T	SHFF *	FF	SHFF	FF	SHFF	FF
5	50	21.251	19.976	26.229	5.725	27.323	13.766
	100	36.030	34.367	58.955	5.605	69.692	46.440
	500	12.858	12.066	65.362	3.132	58.281	45.287
	1000	13.421	12.169	78.276	3.152	66.986	30.674
10	50	56.238	51.780	82.706	0.355	78.215	47.120
	100	57.375	53.294	88.677	1.596	86.230	62.219
	500	39.860	36.808	86.681	6.667	85.319	59.022
	1000	31.370	29.756	89.182	2.775	86.310	68.711
30	50	72.419	67.932	85.041	1.307	85.263	56.602
	100	66.897	61.873	90.000	0.441	90.770	66.561
	500	54.593	49.125	91.742	1.333	87.463	60.769
	1000	37.737	34.025	91.645	0.023	89.271	60.661

* SHFF denotes the percentage relative improvement of the LIS relative to the sample estimator, and FF denotes the percentage relative improvement of the LIS relative to linear shrinkage (similar for other tables).

**Table A2:** Improvements in the five-factor GIS over sample and linear shrinkage in higher-order moment estimation.

		Covariance		Coskewness		Cokurtosis	
N	T	SHFF	FF	SHFF	FF	SHFF	FF
5	50	20.123	18.807	26.228	5.725	26.629	12.907
	100	35.027	33.327	58.954	5.605	69.495	46.078
	500	12.670	11.907	65.363	3.132	58.269	45.273
	1000	13.452	12.120	78.276	3.152	66.987	30.677
10	50	52.666	47.749	82.706	0.355	77.521	45.384
	100	54.901	50.567	88.677	1.596	85.970	61.442
	500	39.043	35.949	86.681	6.067	85.286	58.958
	1000	30.933	29.308	89.181	2.775	86.299	68.656
30	50	68.412	63.234	85.041	1.306	84.768	54.946
	100	63.043	57.347	90.000	0.441	90.486	65.516
	500	52.802	47.077	91.742	1.333	87.368	60.549
	1000	36.319	32.515	91.645	0.036	89.243	60.494

**Table A3:** Improvements in the three-factor LIS over sample and linear shrinkage in higher-order moment estimation.

		Covariance		Coskewness		Cokurtosis	
N	T	SHFF	FF	N	T	SHFF	FF
5	50	50.946	48.781	77.322	25.756	79.708	51.304
	100	43.288	40.547	78.450	22.832	80.752	47.774
	500	30.385	28.988	78.177	29.389	79.146	44.833
	1000	32.580	31.876	82.406	31.083	81.052	35.947
10	50	59.787	56.125	85.540	24.908	82.285	41.463
	100	61.728	58.727	86.175	24.118	88.345	46.467
	500	39.475	37.127	90.641	7.453	87.725	59.601
	1000	39.336	37.850	91.591	7.944	90.486	66.266
30	50	61.356	57.125	85.540	26.908	84.356	43.562
	100	63.135	59.634	86.175	24.118	89.754	48.291
	500	57.077	55.666	93.118	28.627	93.043	54.381
	1000	43.199	41.987	91.340	22.994	91.003	91.023

**Table A4:** Improvements in the three-factor QIS over sample and linear shrinkage in higher-order moment estimation.

		Covariance		Coskewness		Cokurtosis	
N	T	SHFF	FF	N	T	SHFF	FF
5	50	46.830	44.391	77.322	25.756	79.199	50.203
	100	40.626	37.746	78.450	22.832	80.589	47.341
	500	29.589	28.173	78.177	29.389	79.134	44.811
	1000	32.187	31.479	82.406	31.083	81.051	35.944
10	50	52.571	48.119	85.540	24.908	81.478	38.782
	100	56.452	53.073	86.175	24.118	88.188	45.919
	500	37.744	35.328	90.641	7.453	87.695	59.529
	1000	38.393	36.884	91.591	7.944	90.479	66.251
30	50	54.423	49.923	85.540	24.908	83.126	39.302
	100	57.651	55.253	86.175	24.118	89.562	46.782
	500	55.808	54.356	93.118	28.627	93.028	54.338
	1000	42.316	41.086	91.340	22.994	90.997	51.327

**Table A5:** Improvements in the three-factor GIS over sample and linear shrinkage in higher-order moment estimation.

		Covariance		Coskewness		Cokurtosis	
N	T	SHFF	FF	N	T	SHFF	FF
5	50	48.580	46.270	77.322	25.756	9.390	50.651
	100	41.987	39.178	78.450	22.832	80.672	47.561
	500	30.244	28.843	78.177	29.389	79.144	44.829
	1000	32.593	31.889	82.406	31.083	81.052	35.947
10	50	55.680	51.549	85.540	24.908	81.813	39.934
	100	58.846	55.634	86.175	24.118	88.248	46.146
	500	38.636	36.256	90.641	7.453	87.710	59.654
	1000	39.931	37.435	91.591	7.944	90.483	66.260
30	50	57.120	53.342	85.540	24.908	82.602	40.624
	100	59.924	56.201	86.175	24.118	89.417	47.563
	500	56.468	55.037	93.118	28.627	93.035	54.357
	1000	42.820	41.600	91.340	22.994	91.000	51.335
